# Support Vector Machine for Analyzing Contributions of Brain Regions During Task-State fMRI

**DOI:** 10.3389/fninf.2019.00010

**Published:** 2019-03-06

**Authors:** Mengyue Wang, Chunlin Li, Wenjing Zhang, Yonghao Wang, Yuan Feng, Ying Liang, Jing Wei, Xu Zhang, Xia Li, Renji Chen

**Affiliations:** ^1^Beijing Key Laboratory of Fundamental Research on Biomechanics in Clinical Application, School of Biomedical Engineering, Capital Medical University, Beijing, China; ^2^Beijing Stomatological Hospital, Capital Medical University, Beijing, China; ^3^Beijing Institute of Technology, Beijing, China

**Keywords:** generalized linear models, support vector machine, contribution of brain region, task fMRI, lasso regression

## Abstract

The mainstream method used for the analysis of task functional Magnetic Resonance Imaging (fMRI) data, is to obtain task-related active brain regions based on generalized linear models. Machine learning as a data-driven technical method is increasingly used in fMRI data analysis. The language task data, including math task and story task, of the Human Connectome Project (HCP) was used in this work. We chose a linear support vector machine as a classifier to classify math and story tasks and compared them with the activated brain regions of a SPM statistical analysis. As a result, 13 of the 25 regions used for classification in SVM were activated regions, and 12 were non-activated regions. In particular, the right Paracentral Lobule and right Rolandic Operculum which belong to non-activated regions, contributed most to the classification. Therefore, the differences found in machine learning can provide a new understanding of the physiological mechanisms of brain regions under different tasks.

## Introduction

In functional magnetic resonance data analysis, GLM (generalized linear models) are one of the most common model-based methods that correlate measured hemodynamic signals with controlled experimental variables (Friston et al., 1994; Holmes and Friston, 1998). Specifically, each voxel of the functional Magnetic Resonance Imaging (fMRI) image and the experimental paradigm are analyzed by a generalized linear model, and each voxel corresponds to a coefficient Bata of a regression equation, and all coefficients are combined to form a statistical parameter map (Yan et al., 2011; Wu et al., 2012). In a group analysis, a one sample *t*-test is performed on the statistical parameter maps of all subjects to determine the activation region of the group (Beckmann et al., 2003). Although the GLM is currently the dominant approach to brain activation detection, there is growing interest in multivariate approaches (Zhang et al., 2009). For example, machine learning as a data-driven technology is not only sensitive to subtle spatial differentiation patterns, but also capable of exploring the inherent multivariate nature of high-dimensional image data (Norman et al., 2006). Since machine learning can find features that contribute most to classification (Meier et al., 2012; Lv et al., 2015), differences found can provide a new understanding of the physiological mechanisms of brain regions under different tasks.

Applying machine learning methods to neuroimaging data began with the work of Haxby et al. (2001), who recognized the distribution characteristics of visual cortex activation patterns from functional MRI. At present, machine learning has been widely used in fMRI data classification (Yan et al., 2017a,b) to explore the cognitive state of the brain (Yan et al., 2018). Under different visual stimulation conditions, the stimulus may be different visual pictures (objects or people, shoes or bottles), raster stimulation at different angles, etc., and the type of task received by the subject is determined by classifying the collected fMRI data (Haxby et al., 2001; Kamitani and Tong, 2005; Norman et al., 2006). Machine learning is used in psychiatry to distinguish patients from controls. Patients with severe depression (Fu et al., 2008) were classified with an accuracy rate of 70 to 80%. Individuals and controls with autism spectrum disorder were distinguished based on two fMRI experiments (Chanel et al., 2016). Machine learning is therefore a promising method used to detect brain state (Ecker and Murphy, 2014). Machine learning mostly uses support vector machines as classifiers in functional magnetic resonance data classification (De et al., 2008; Pereira et al., 2009; Ecker et al., 2010; Xin et al., 2013).

When the number of features far exceeds the number of subjects, it will cause problem which commonly occurs in machine learning known as the curse of dimensionality (Bellman, 1961). If the dimension reduction of features cannot be performed, it is easy to cause over-fitting (Guyon, 2003). Over fitting means that the model has poor generalization ability, that is, the ability to accurately predict new samples is poor (Mayer et al., 2009). Therefore, feature selection is required before training the model (De et al., 2007; Pereira et al., 2009; Mwangi et al., 2014).

In this study, we sought to explore the effects of activated brain regions and inactivated brain regions on the classification results of functional magnetic resonance data for different tasks. We extracted the average *t* value of the generalized linear model as the eigenvector and chose the Lasso regression algorithm (Tibshirani, 1996) for feature dimension reduction. Using a linear support vector machine, the classification weight was used as an index to evaluate the importance of each brain region in the classification and compared this with the group analysis results. Results revealed two brain regions that did not appear in the activated brain region but contributed significantly to the classification, namely the right Paracentral Lobule and the right Rolandic Operculum.

## Materials and Methods

### Participants

Experimental data for 1046 healthy subjects was obtained from the open source database, WU-Minn Human Connectome Project (HCP) Data - 1200 Subjects (HCP_1200), published by the Public Connectome Data^1^. Most participants were between the ages of 22 and 35. All participants had no previously documented history of psychiatric, neurological or medical disorders that affected their brain function. Of the 1046 participants, 560 were female and 486 were male, 223 were between the ages of 22–25, 455 were between the ages of 26–30, 357 were between the ages of 31–35 and 11 were over the age of 36. We used the 3T MR Language Task fMRI Preprocessed sessions.

### Experimental Paradigms

The language task contained an auditory story presentation with comprehension questions and math problems. It consisted of two runs that each had eight blocks (four story blocks and four math blocks) randomly combined. The length of each block varied, but the average length was about 30 s. In order to complete a 3.8 min run, the math task blocks needed to match the length of the story task blocks, and additional math tasks were added when the total length was less than 3.8 min. The story blocks presented participants with a brief auditory story (around 5–9 sentences) adapted from a collection of Aesop’s fables. After each story, the participant was asked about the topic of the story, in the form of a 2-alternative forced-choice question. For example, after a story about an eagle that saves a man who had done him a favor, participants were asked, “Was that about revenge or reciprocity?” Participants pressed a button under the right index finger to select the first choice or a button under the right middle finger to select the second choice. Math tasks were also presented in a phonetic manner, requiring participants to complete simple addition and subtraction problems. Each series of arithmetic operations ended with the word “equals” followed by two alternative choices, e.g., “Four plus twelve, minus two plus nine, equals twenty-two or twenty-three?” The participants pushed a button to select either the first or the second answer (Binder et al., 2011; Barch et al., 2013).

### fMRI Data Acquisition

Whole-brain EPI acquisitions were acquired with a 32 channel head coil on a modified 3T Siemens Skyra with TR = 720 ms, TE = 33.10 ms, flip angle = 52°, BW = 2290 Hz/Px, in-plane FOV = 208 × 180 mm, 72 slices, 2.0 mm isotropic voxels, with a multi-band acceleration factor of 8 (Feinberg et al., 2010; Moeller et al., 2010). For further information please refer to Ugurbil et al. (2013) for an overview of the acquisition details of the task fMRI. Two runs of each task were acquired, one with a right-to-left phase encoding and the other with a left-to-right phase encoding.

### fMRI Data Processing

#### Preprocessing

**FIGURE 1:**
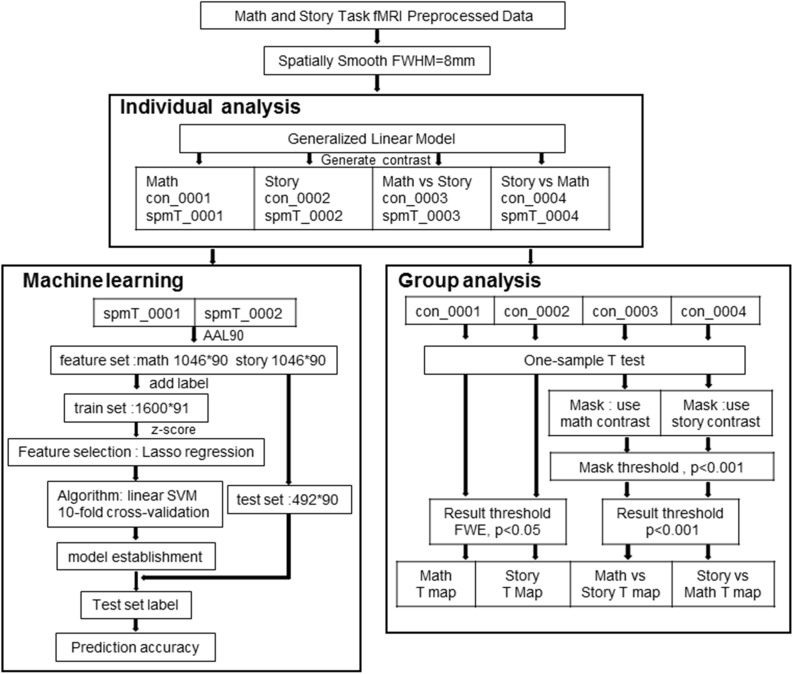
Data processing flowchart for SPM and machine learning analysis.

We used the 3T MR Language Task fMRI Preprocessed data. This data was processed using FSL and FreeSurfer. The steps included gradient unwarping, motion correction, fieldmap-based EPI distortion correction, brain-boundary-based registration of EPI to a structural T1-weighted scan, non-linear (FNIRT) registration into MNI152 space, and grand-mean intensity normalization. In addition, spatial smoothing was done with an 8 mm full-width at half-maximum Gaussian core (Figure 1) for GLM analysis.

### SPM Statistical Analysis

In order to identify the differences between the two tasks and to evaluate the significance of functional activation, we used a GLM analysis. In the first level (within-subject) analysis, the data was skillfully modeled in GLM. Four kinds of contrast images were created for each participant, including math task, story task, math vs. story task and story vs. math task. In the second-level analysis, the contrast (con files) images were used from the first-level analyses of all 1046 subjects. The four conditions were analyzed by one-sample *t*-test analysis. The SPM (T) map of math and story tasks were obtained and the threshold was *p* < 0.05(FWE) at voxel level. To eliminate artifacts, we used math contrasts and story contrasts as a mask and the mask threshold was *p* < 0.001 at voxel level for math vs. story and story vs. math tasks, respectively. The SPM (T) map of math vs. story and story vs. math tasks were then obtained and the threshold was *p* < 0.05(FWE) at voxel level. These results were used to analyze the activation of brain functions and were compared with the results of machine learning.

### Classification Using Machine Learning

After the SPM^2^ processed individual data, the spmT file was generated for each of the two experimental conditions. Under GRETNA (Wang et al., 2015), the AAL90^3^ (Anatomical Automatic Labeling) template was used to segment the brain region of the spmT file, and the average statistical *T* value of each brain region was extracted to generate a 90 × 1 feature matrix. For a total of 1046 participants, the feature vector was: math task 1046 × 90, story task 1046 × 90. The characteristics of 800 subjects were selected as a training set. The math task tag was 1, the story task tag was -1 and the training set was sent to the classifier for classification. The remaining 246 subjects were used as the prediction test set. Before classification, a z-score was used to normalize the preprocessed training set. And the Lasso regression algorithm was used for feature selection. Then the linear support vector machine was used as the kernel function and the 10-fold cross-validation was used to calculate the correct rate of training. Brain region contribution results could also be obtained while establishing a classification model. Finally, the test set was sent to the classifier to obtain the classification label and the accuracy of the prediction result was calculated. In order to obtain the optimal classification result, it was necessary to debug the classification parameters to predict the correctness of the results as the debugging standard. It included two parameters, one was the regularization parameter α of the Lasso algorithm, and it directly determined the number of features. The larger the alpha, the sparser the model, therefore, more regression coefficients β were set to 0, thus deleting some features to achieve feature selection. The other was the penalty coefficient C of the linear support vector machine, and it directly determined the accuracy of training. The value of C was generally between 0.01 and 0.1. The contribution of the brain region was proposed under two preconditions: firstly, the feature was extracted based on the region partitioned by the brain template, so that the feature was associated with the three-dimensional brain structure, therefore, each feature corresponded to a brain region; secondly, the linear support vector machine was selected as the classifier, because the weight of the linear support vector machine was in one-to-one correspondence with the feature vector. The larger the weight value, the more important the corresponding feature was to the establishment of the classification decision surface. Through the relationship between the features and the brain regions and the relationship between features and classification weights, the corresponding relationship between brain regions and weights was established. In simple terms, the contribution of the brain region, was the weight value of the optimal decision function, of the linear support vector machine classifier.

## Results

### Behavioral Data

**FIGURE 2:**
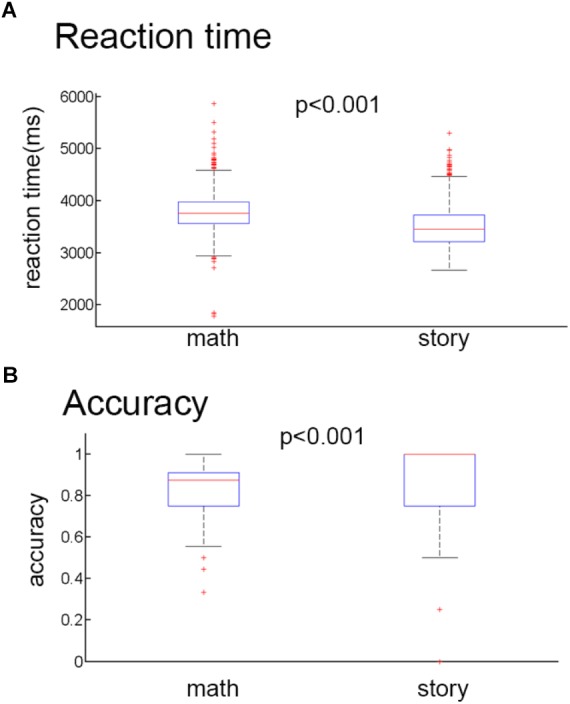
Behavioral results. **(A)** Mean reaction time for the math stimuli and story stimuli. **(B)** Mean accuracy rates for the math stimuli and story stimuli.

The behavioral data were collected from 1046 participants during the fMRI experiments. Only one subject’s data was lost during the experiment. We used the average reaction time and correct rate data of 1045 participants for statistical analysis. There were two tasks. The mean reaction times (RT) (Figure 2A) and the mean accuracy (Figure 2B) were 3.79 ± 0.38 s and 83.28% (SD 3.42), respectively, for the math task and 3.50 ± 0.39 s and 92.57% (SD 12.94), respectively, for the story task. Two tailed two-sample *t*-tests were performed to compare the mean RTs and the mean accuracy between the math task and story task. The results showed that the math task had a slower reaction time compared to the story task (*t* = 17.260, *P* < 0.001). And the accuracy of the math task was significantly lower than the story task (*t* = 15.834, *P* < 0.001).

### Imaging Data

#### Group Analysis Results

**FIGURE 3:**
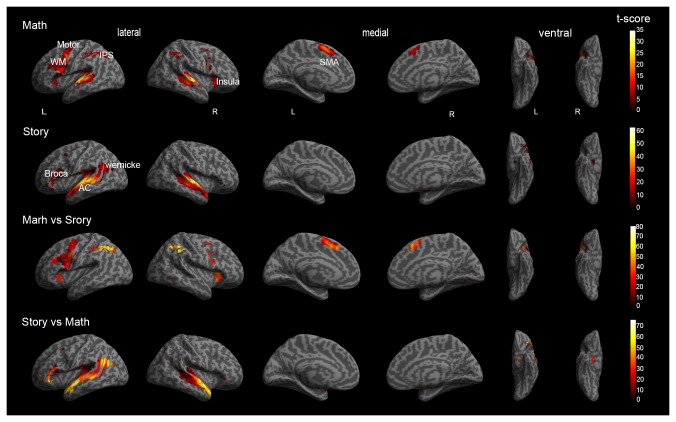
Global brain activation of the group analysis. **(A)** Math shows a three-dimensional brain activation map in the math task. **(B)** Story shows a three-dimensional brain activation map in the story task. **(C)** Math vs. Story shows the difference of activated brain regions between the Math task relative to the Story task. **(D)** Story vs. Math shows the difference of activated brain regions between the Story task relative to the Math task. WM = working memory, IPS = Intraparietal sulcus, AC = Auditory cortex, SMA = Supplementary Motor Area.

The specific group results of the four groups of activated brain regions were shown in Table 1. The activations of math and story tasks showed that both the left and right temporal lobe were activated (Figure 3A,B). In addition to the temporal lobe, in the math task, the brain area with a greater activation intensity included: the left Precentral Gyrus, left Middle Temporal Gyrus, left Superior Temporal Gyrus, right Inferior Frontal Gyrus and the right Middle Frontal Gyrus (Wang et al., 2007). In the story task, the brain area with a greater activation intensity included: the left Inferior Frontal Gyrus, left Middle Frontal Gyrus and the right Inferior Semi-Lunar Lobule. Compared to the story results (Figure 3C), the math results included: the left Inferior Frontal Gyrus, left Inferior Parietal Lobule and the left Superior Parietal Lobule which had a higher activation intensity than the story task; while the Superior Parietal Lobule and Inferior Parietal Lobule only activated in the math task. Compared with the math results (Figure 3D), the brain area of the story task, the left Inferior Temporal Gyrus, Superior Temporal Gyrus and the Middle Temporal Gyrus, had a significantly higher activation intensity than the math task, and the Parahippocampal Gyrus Amygdala on the left and right sides only activated in the story task (Binder et al., 2011; Barch et al., 2013).

**Table 1:** Activated regions during the two auditory stimuli and the different activated regions between them.

Cluster size (voxels)	Anatomical regions and BA (FWE, *p* < 0.05)	*t*-score	x	y	z
**Math**					
2498	L Precentral Gyrus BA 6	20.70	-48	-2	44
	L Precentral Gyrus BA 6	20.22	-44	2	34
	L Middle Frontal Gyrus BA 6	15.03	-26	-4	50
1700	L Superior Temporal Gyrus BA41	33.25	-56	-20	4
	L Superior Temporal Gyrus BA 38	6.26	-56	6	-6
1542	R Superior Temporal Gyrus BA 22	36.82	64	-18	2
1266	L Inferior Parietal Lobule BA 40	18.83	-42	-42	42
	L Superior Parietal Lobule BA 7	10.80	-26	-62	44
	L Precuneus BA 7	10.51	-28	-66	36
857	R Tuber	27.62	30	-60	-28
782	R Inferior Parietal Lobule BA 40	15.51	46	-38	42
733	L Superior Frontal Gyrus BA 6	20.23	-6	10	54
	R Superior Frontal Gyrus BA 8	12.33	8	16	50
459	R Inferior Frontal Gyrus BA 47	18.12	32	26	0
316	R Inferior Frontal Gyrus BA 9	8.83	44	6	30
284	L Uvula	20.54	-28	-64	-26
189	R Middle Frontal Gyrus BA 6	9.16	32	0	52
50	R Substantia nigra	7.35	10	-14	-10
44	R Inferior Semi-Lunar Lobule	13.00	18	-68	-44
23	R Inferior Temporal Gyrus BA 20	7.93	54	-48	-8
23	L Caudate-Caudate Head	5.56	-12	6	4
22	R Superior Parietal Lobule BA 7	6.54	12	-66	56
17	L Substantia nigra	5.80	-8	-16	-12
9	R Lingual Gyrus BA 18	5.10	8	-86	-2
6	L Thalamus	4.71	-10	-14	2
4	L Postcentral Gyrus BA 3	4.65	-36	-26	50
**Story task**					
4087	L Superior Temporal Gyrus BA 22	59.72	-62	-16	4
	L Middle Temporal Gyrus BA 21	32.53	-56	4	-10
	L Superior Temporal Gyrus BA 38	31.59	-52	10	-16
2768	R Superior Temporal Gyrus BA 22	63.16	62	-12	2
	R Superior Temporal Gyrus BA 38	26.30	48	12	-24
759	L Inferior Frontal Gyrus BA 47	25.10	-48	30	-6
	L Inferior Frontal Gyrus BA 45	19.17	-52	22	16
442	R Inferior Semi-Lunar Lobule	22.01	22	-74	-36
	R Pyramis	21.03	20	-72	-28
	R Culmen	6.40	30	-60	-26
110	L Middle Frontal Gyrus BA 6	9.82	-42	4	48
71	R Parahippocampal Gyrus Amygdala	10.06	18	-6	-14
37	L Parahippocampal Gyrus Amygdala	7.79	-18	-8	-14
33	R Inferior Frontal Gyrus BA 47	8.40	46	32	-8
11	R Cerebellar Tonsil	8.68	6	-56	-42
9	L Postcentral Gyrus BA 3	5.56	-36	-26	50
9	R Superior Temporal Gyrus BA 39	4.67	52	-54	22
**Math vs. Story**					
2717	L Insula BA 13	50.5	-34	18	6
	L Inferior Frontal Gyrus BA 6	47.82	-44	2	32
	L Sub-Gyral BA 6	46.54	-26	4	56
1529	L Inferior Parietal Lobule BA 40	74.98	-42	-46	44
	L Superior Parietal Lobule BA 7	62.76	-28	-64	46
	L Superior Parietal Lobule BA 7	51.08	-10	-68	52
1337	R Insula BA 13	59.33	40	18	0
	R Middle Frontal Gyrus BA 6	53.07	32	4	56
	R Inferior Frontal Gyrus BA 9	41.46	46	6	28
1026	R Inferior Parietal Lobule BA 40	80.95	50	-40	48
	R Superior Parietal Lobule BA 7	61.34	32	-64	46
	R Superior Parietal Lobule BA 7	48.52	12	-68	52
884	R Medial Frontal Gyrus BA 8	61.51	4	20	46
866	R Cerebellar Tonsil	48.22	32	-58	-32
	R Declive	24.72	10	-74	-22
344	L Uvula	46.25	-32	-64	-26
	L Declive	31.49	-12	-76	-22
139	R Caudate Body	16.24	18	2	16
	R Substantia nigra	10.12	10	-14	-10
101	L Substantia nigra	12.18	-6	-16	-14
	L Thalamus Medial Dorsal Nucleus	9.36	-10	-18	10
	L Thalamus	7.18	-12	-12	2
76	L Lentiform Nucleus Putamen	14.76	-20	2	16
	L Nucleus Medial Globus Pallidus	6.76	-12	0	-2
	L Lentiform Nucleus Putamen	6.39	-14	8	2
49	R Inferior Temporal Gyrus BA 20	46.97	54	-46	-10
	R Middle Temporal Gyrus BA 20	6.11	50	-38	-6
45	R Inferior Semi-Lunar Lobule	19.35	18	-68	-44
32	R Lingual Gyrus BA 17	14.26	8	-84	2
**Story vs. Math**					
4175	L Inferior Temporal Gyrus BA 21	77.75	-58	-6	-12
	L Superior Temporal Gyrus BA 38	70.16	-48	10	-26
	L Middle Temporal Gyrus BA 39	62.72	-50	-62	24
2764	R Superior Temporal Gyrus BA 38	68.2	46	12	-28
	R Middle Temporal Gyrus BA 21	67.72	54	-4	-14
	R Insula BA 13	22.56	40	-24	16
646	L Inferior Frontal Gyrus BA 47	63.66	-44	30	-12
	L Inferior Frontal Gyrus BA 45	42.05	-54	26	10
279	R Pyramis	61.07	26	-76	-34
104	R Parahippocampal Gyrus Amygdala	41.73	20	-4	-16
59	R Superior Temporal Gyrus BA 39	38.66	54	-58	22
55	L Parahippocampal Gyrus Amygdala	50.85	-20	-6	-18
51	R Middle Frontal Gyrus BA 11	42.62	44	34	-12
	R Inferior Frontal Gyrus BA 47	33.73	50	32	-6
44	L Middle Frontal Gyrus BA 6	18.33	-40	10	50
20	R Cerebellar Tonsil	26.82	6	-56	-42


#### Parameter Debugging Result

**FIGURE 4:**
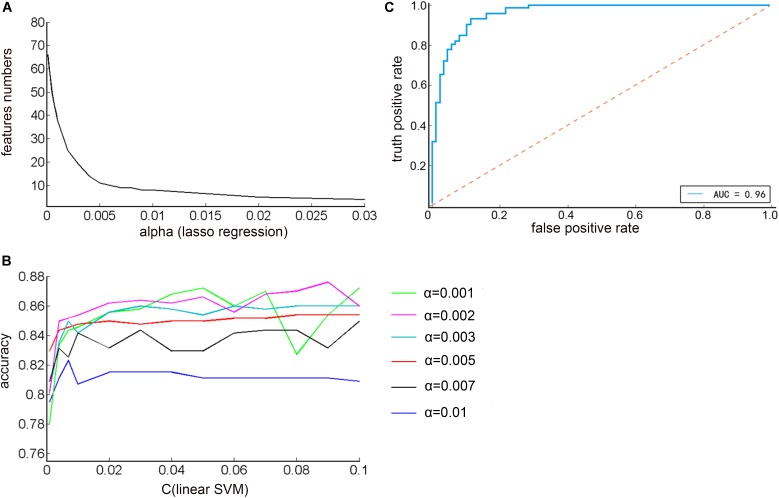
**(A)** The relationship between the regularization parameter alpha of the Lasso regression algorithm and the number of feature selections **(B)** The relationship between the penalty coefficient C of the linear support vector machine and the correct rate of the prediction result under different alpha values **(C)** ROC curve of optimal classification results.

As shown in Figure 4A, it was found that as the α increased, the number of features decreased exponentially. Therefore, in order to reduce the dimensional disaster and improve the classification performance of the classifier, the appropriate number of important features were selected, α were taken as: 0.001, 0.002, 0.003, 0.005, 0.007, 0.01, and the corresponding feature numbers were: 38, 25, 19, 11, 9, 8. Next, the penalty coefficient C of the linear support vector machine was debugged, and finally the accuracy of the prediction result was used as a criterion for evaluating the performance of the classifier. As shown in Figure 4B, when α = 0.002, C = 0.09, the highest classification accuracy rate was 87.60%. The current parameters and the effects of the trained models could be visually evaluated by plotting the ROC curve and the AUC indicator. As shown in Figure 4C, the area under the curve was 0.96, which was close to 1, indicating that the classifier had a good classification effect.

#### Machine Learning Results

**FIGURE 5:**
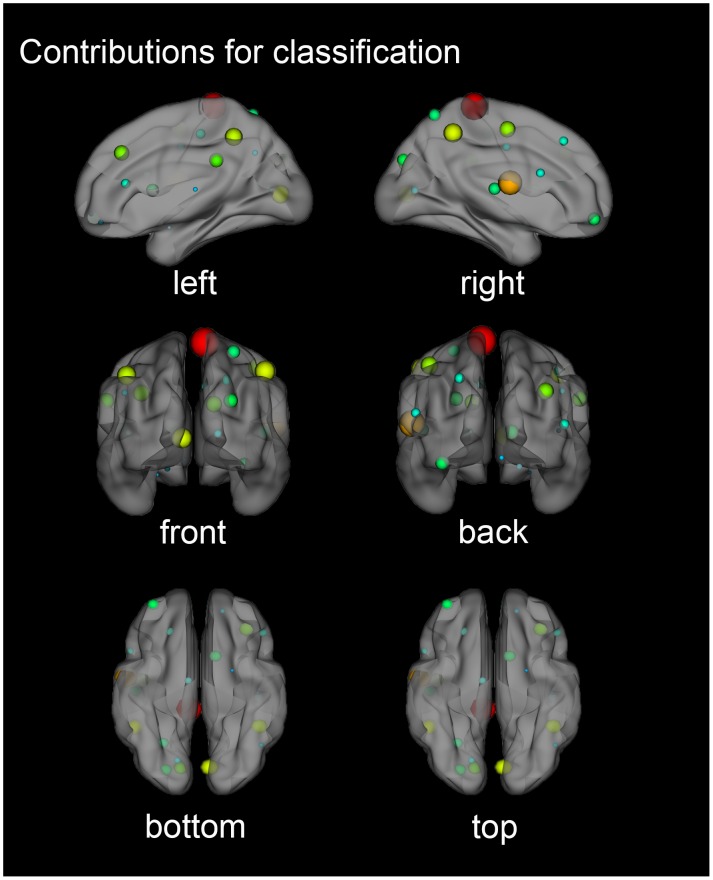
Three-dimensional contribution of brain regions for classification. Each node represented a brain region divided by AAL90 (Anatomical Automatic Labeling template). The node colors represent different regions and the node size was scaled according to the weight value of the brain regions. The greater the contribution of the brain region, the larger the radius of the node.

As shown in Figure 5, a three-dimensional brain region contribution distribution map in six directions was shown. Some regions tended to exhibit higher classification weights than others. In particular, if the weight of some areas was at least greater than the average weight of all areas, plus a standard deviation of one time, we considered these areas to have significant weights (Tian et al., 2011). The mean value plus the standard deviation of the contribution was equal to 0.0614. The brain region with a contribution greater than 0.0614 was considered significant, including: the right Paracentral Lobule, right Rolandic Operculum and the right Inferior Parietal Lobule, excluding the supramarginal and angular gyri.

Comparing the classified brain region contribution results and the group analysis activation region results, as shown in Table 2, it was found that 13 of the 25 characteristic brain regions overlapped with the group analysis activated brain regions. Among the 13 brain regions, there were 11 brain regions that overlapped with a different activation map between the math task and the story task. The 11 brain regions were: the left and right Inferior Parietal Lobe (not include supramarginal and angular gyri), left and right Middle Frontal Gyrus, left Supramarginal Gyrus, right Superior Parietal Gyrus, right Superior Frontal Gyrus, dorsolateral, right Inferior Frontal Gyrus, opercular part, right Angular Gyrus, left Amygdala, left Heschl Gyrus. Moreover, these coincident regions had strong activation in the group analysis results (*t* values were greater than 18). The remaining 12 brain regions did not overlap with the group analysis activation region results, including the two brain regions with significant contributions: the right Paracentral Lobule and right Rolandic Operculum.

**Table 2:** Comparison with degrees between the brain region contribution and group analysis: Label and regions represent the brain region label and brain region name of the classification result under the AAL90 template.

Label	Region	Cs	Coincidence brain region	M(*t*)	S(*t*)	M vs. S(*t*)	S vs. M(*t*)
70	Paracentral_Lobule_R	0.0918	None				
18	Rolandic_Oper_R	0.0792	None				
62	Parietal_Inf_R	0.0618	R Inferior Parietal Lobule			80.95	
43	Calcarine_L	0.061	None				
61	Parietal_Inf_L	0.0592	L Inferior Parietal Lobule			74.98	
2	Precentral_R	0.055	None				
7	Frontal_Mid_L	0.0529	L Middle Frontal Gyru	15.03	9.82		18.33
46	Cuneus_R	0.0522	None				
63	SupraMarginal_L	0.048	L Inferior Parietal Lobule			74.98	
50	Occipital_Sup_R	0.0449	None				
71	Caudate_L	0.0431	None				
60	Parietal_Sup_R	0.0425	R Superior Parietal Lobule			61.34	
10	Frontal_Mid_Orb_R	0.0409	R Middle Frontal Gyrus			53.07	
80	Heschl_R	0.0394	None				
57	Postcentral_L	0.0318	L Postcentral Gyrus	4.65	5.56		
4	Frontal_Sup_R	0.0314	R Middle Frontal Gyrus			53.07	
12	Frontal_Inf_Oper_R	0.0308	R Inferior Frontal Gyrus		8.40	41.46	33.73
34	Cingulum_Mid_R	0.0268	None				
44	Calcarine_R	0.0246	None				
65	Angular_L	0.0195	R Insula			59.33	22.56
5	Frontal_Sup_Orb_L	0.0177	L Superior Frontal Gyrus		4.44		
25	Frontal_Mid_Orb_L	0.0152	None				
41	Amygdala_L	0.0121	L Parahippocampal Gyrus		7.79		50.85
79	Heschl_L	0.011	L Insula			50.50	
19	Supp_Motor_Area_L	0.0073	None				


*The order of the table is arranged by the brain region contribution degree from large too small. Cs represents the contribution score of the brain region. The coincident brain region indicates the activated brain region in which the classification result coincides with the group analysis solution. The following sections showed the groups and t values that appear in the coincident brain regions: math, story, math vs. story, story vs. math.*

*^∗^R = right, L = left, Oper = operculum, Inf = Inferior, Mid = Middle, Sup = Superior, Orb = orbital part, Supp = Supplementary.*

## Discussion

One of the experimental paradigms designed by Wang et al. was the auditory computing task in Mandarin Chinese and English. The calculation included addition and multiplication. It is similar to the math task. Study participants included 19 adult native Mandarin Chinese speakers, with no history of speech or hearing impairments. The active brain regions of the calculation task in English after the group analysis include: the left Precentral Gyrus, left Middle Temporal Gyrus, right Inferior Frontal Gyrus, and the right Middle Frontal Gyrus (Wang et al., 2007). Barch et al. (2013) chose 77 participants (58 women and 19 men) and all participants were aged between 22 and 35, with no previously documented history of psychiatric, neurological or medical disorders that are known to influence brain function. Binder et al. (2011) chose 34 healthy, right-handed adults as participants. (17 women and 17 men), aged between 18 and 50 years (mean 29 years). They all used the same experimental paradigm of this article, and similar results were obtained: the story vs. math results showed that the largest activation cluster involved the temporal lobe and strong medial temporal activation involved the uncus, amygdala, and the anterior hippocampus, extending posteriorly into the parahippocampal and posterior fusiform gyrus.

### Comparative Analysis of Brain Region Contribution and Group Results

The contribution of brain regions is to combine the different partitions of the three-dimensional physiological structure in the brain space, with the weights of the classifiers. Therefore, the brain region contribution degree reflects the importance of different brain regions to the classification results. The higher the contribution value is, the more important the brain area is for classification results. Classification is to compare the differences between the two categories. Therefore, the results of the classification mostly coincided with the differential activation of the brain region. These overlapping brain regions were: the Middle Frontal Gyrus, which is involved in expressive language processes including semantics (Brown et al., 2010), grammar and syntax. Broca’s area played a role in syntactic processing during Chinese reading comprehension, verbal fluency (Abrahams et al., 2003), and verbal working memory (Leung et al., 2002). Inferior Parietal Lobule has been involved in the perception of emotions, facial stimuli and interpretation of sensory information. The left Supramarginal Gyrus was most likely involved with language perception and processing (Gazzaniga et al., 2013). The left Heschl Gyrus, which is found in the area of the primary auditory cortex buried within the lateral sulcus of the human brain, was the first cortical structure to process incoming auditory information. The Heschl Gyrus was active during auditory processing under fMRI for tone and semantic tasks (Warrier et al., 2009). The right Superior Frontal Gyrus, dorsolateral, is involved in self-awareness, in coordination with the action of the sensory system (Goldberg and Harel, 2006; Wang et al., 2017). The Amygdala plays a major role in memory, decision making, and emotional response (including fear, anxiety, and aggression), which is thought to be part of the limbic system (Amunts et al., 2005). The left Amygdala, plays a major role in memory, decision making, and emotional response (including fear, anxiety, and aggression), which is thought to be part of the limbic system (Amunts et al., 2005). Moreover, the intensity of activation of these overlapping brain regions in the results of the group analysis reflected the correctness of the classification features and could identify brain regions with large activation differences between the two tasks.

**FIGURE 6:**
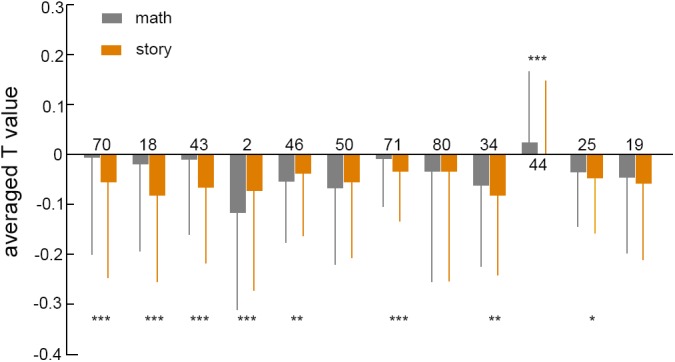
The averaged *T* value in inactivated brain regions under two tasks. The numbers on the 12-column chart represented the brain area number of the AAL90 template, the gray box represented the math task, and the orange represented the story task. The number of asterisks represented the degree of *p* value. ^∗^*p* < 0.05, ^∗∗^*p* < 0.01, ^∗∗∗^*p* < 0.001.

There were 12 brain regions in the feature brain region that did not coincide with the group activation results, including two brain regions with significant contributions: the right Paracentral Lobule, which is concerned with Motor and sensory innervations of the contralateral lower extremity (Spasojević et al., 2013) and it is also responsible for control of defecation and urination, and the right Rolandic Operculum. Some studies have proven that articulatory disorders correspond with lesions of the Rolandic Operculum (Tonkonogy and Goodglass, 1981). The reason for the significant difference between the classification result and the group analysis result can be explained by using the Paracentral Lobule brain area as an example. On the one hand, when comparing the brain regions of the two task differences in the group analysis, a mask (Gajdoš et al., 2016) was added to eliminate the pseudo activation. The mask was defined by the activation of the brain area of the math or story task. As shown in Figure 6, the *T* value of the brain region (label number 70) was negative for both tasks. Therefore, the differential activation of the brain area must be included in the scope of the single task activation brain area. The main function of the Paracentral Lobule brain area is to control the movement of the contralateral lower limbs and sensory innervation. The functionality of the Paracentral Lobule was independent of the activation of the task and was not activated in the separate analysis of math and story tasks. Therefore, the differential brain regions of the two tasks were unlikely to show activation in the Paracentral Lobule brain region. On the other hand, from the classification principle (Cherkassky, 1997), machine learning did not need to consider the problem of pseudo activation. The selection of features was not limited to the activation range, but the whole brain range. The linear support vector machine mapped the feature vector from the Euclid space to the Hilbert space, making the data set linearly separable in the high-dimensional space. In Hilbert space, finding such a decision surface, not only separated the two types of features, but also made the distance between the two types of features, to this decision surface, as large as possible (Schölkopf, 2000; Huang et al., 2012). The greater the distance between the two types of features, the greater the weight of the classifier, and the greater the contribution value of the brain region corresponding to the feature. Therefore, the contribution essentially reflected the difference between the two types of features corresponding to the brain region in the Hilbert space. The Paracentral Lobule brain region had the highest contribution, indicating that the distance between the corresponding features of the brain region was very far in the high-dimensional space. We speculated that the difference in this brain region was not obvious in low-dimensional space, and statistical analysis did not show any significance.

Machine learning used the difference between the two tasks for classification. Among the negatively activated brain regions, the difference was more obvious, so the contribution in classification was higher than that in the activated brain region. However, the mechanism of these negatively activated brain regions in task execution remains unclear. This is because, in the two tasks used within the brain regions involved, the mechanism was quite different from the mechanism for negatively activating the brain region, therefore, there was no need to use negative activation brain regions for task execution. Depending on the supply of cerebral blood flow, the higher the degree of correlation of the regional function, the greater the degree of cerebral blood flow supply.

We compared the *T* values of 12 inactive brain regions for two tasks, as shown in Figure 6. The *T* values of brain regions in both tasks were mostly negative, and the paired sample *t*-test mostly had a *p* value of less than 0.05. This showed that there was a significant difference between the two tasks in the negative activation of brain regions. The negative activation of brain regions varied greatly among different tasks, suggesting that in addition to activating brain regions, negative activation of brain regions played an important role in brain research.

In order to study the contribution of the brain region to the classification, the linear support vector machine was selected as the classifier, because the weight value of the classifier reflected the importance of the feature to the classifier. In addition, Lasso regression was selected as the feature selection method, which was related to the training of the final machine learning algorithm model. The training model was trained based on the input training data. After the training was completed, the features were sorted based on the model representation and the importance of the features. It was only a screening process. If a feature has a strong influence on the classification performance, it will be retained, and will be zero if it has no effect on the classifier. This method did not change the correspondence between brain regions and features.

## Conclusion

In this paper, the average *T* value of the one-sample generalized linear model was extracted as the eigenvector. The Lasso regression algorithm and the linear support vector machine were used for classification, and the result was compared with the SPM group analysis activation result. It was found that there were coincident brain regions and non-coincident brain regions: the coincident brain regions were mostly the difference between tasks to activate the brain regions, and the activation intensity was strong. Non-coincident brain regions included brain regions with significant classification contributions, right Paracentral Lobule and right Rolandic Operculum. The difference between the two results was mainly due to the difference in the algorithm. In the statistical analysis, in order to eliminate pseudo-activation, the differential activation was limited to a single task activation range; while machine learning did not need to consider pseudo-activation, which can be from the scope of the whole brain, it found feature brain regions that were not related to task activation but contributed significantly to classification. In summary, the contribution of the brain region was from another perspective, analyzing the difference between the two states of brain activity, and finding important brain regions with no statistical difference. This suggested an important role for negative activation of brain regions in brain research.

## Data Availability

Publicly available datasets were analyzed in this study. This data can be found here: https://db.humanconnectome.org/.

## Author Contributions

MW, CL, JW, YL, XZ, and XL analyzed the data using SPM. MW, WZ, RC, YW, and YF analyzed the data using machine learning. MW and CL prepared the figures, and drafted the manuscript. WZ and RC contributed substantial to wrote and revised the manuscript. All authors contributed to manuscript development, and read and approved the final manuscript.

## Conflict of Interest Statement

The authors declare that the research was conducted in the absence of any commercial or financial relationships that could be construed as a potential conflict of interest.

## Footnotes

1 https://db.humanconnectome.org/

2 https://www.fil.ion.ucl.ac.uk/spm

3 http://www.gin.cnrs.fr/en/tools/aal-aal2/
